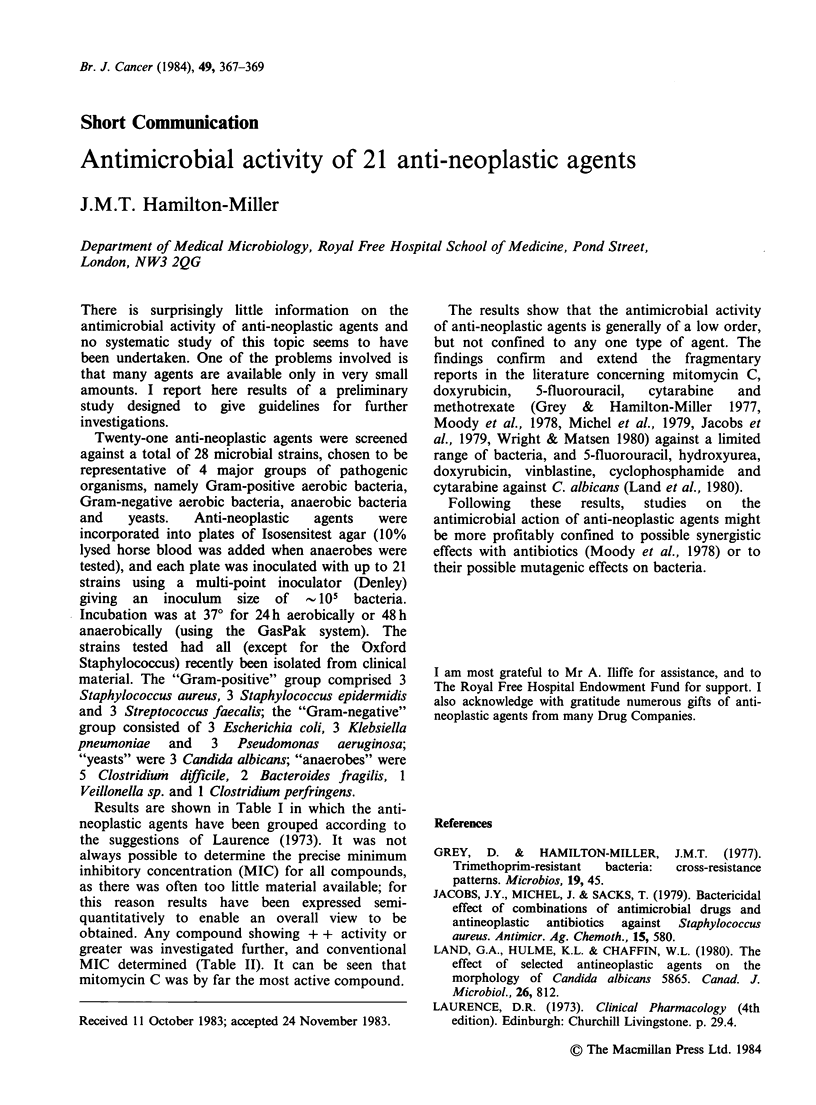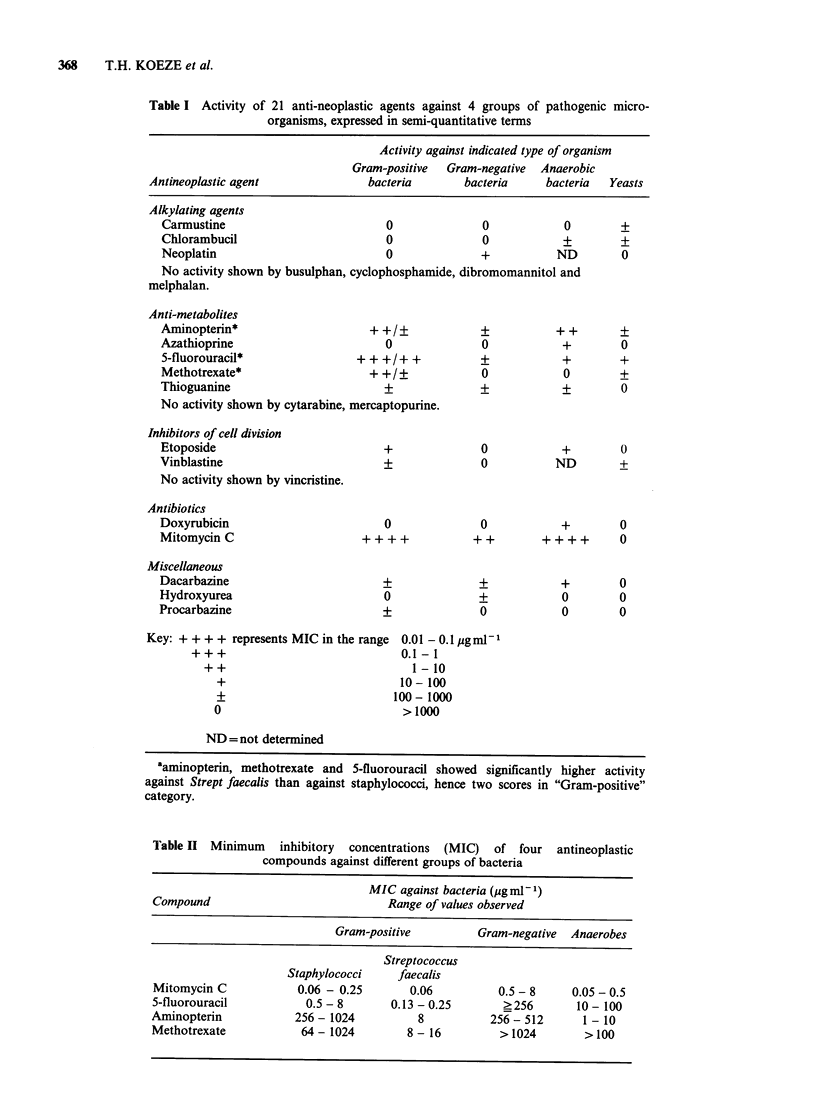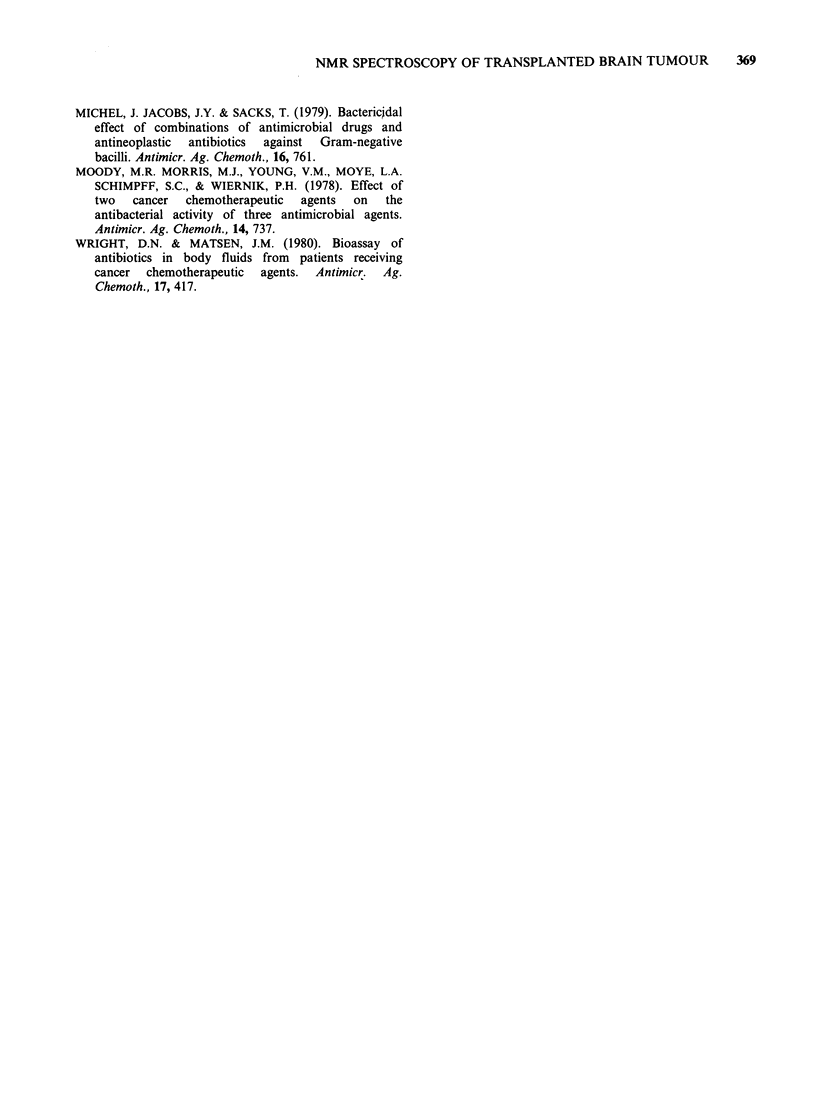# Antimicrobial activity of 21 anti-neoplastic agents.

**DOI:** 10.1038/bjc.1984.58

**Published:** 1984-03

**Authors:** J. M. Hamilton-Miller


Br. J. Cancer (1984), 49, 367–369

### Short Communication

### Antimicrobial activity of 21 anti-neoplastic agents

J.M.T. Hamilton-Miller

*Department of Medical Microbiology, Royal Free Hospital School of Medicine, Pond Street, London, NW3 2QG*

There is surprisingly little information on the antimicrobial activity of anti-neoplastic agents and no systematic study of this topic seems to have been undertaken. One of the problems involved is that many agents are available only in very small amounts. I report here results of a preliminary study designed to give guidelines for further investigations.

Twenty-one anti-neoplastic agents were screened against a total of 28 microbial strains, chosen to be representative of 4 major groups of pathogenic organisms, namely Gram-positive aerobic bacteria, Gram-negative aerobic bacteria, anaerobic bacteria and yeasts. Anti-neoplastic agents were incorporated into plates of Isosensitest agar (10% lysed horse blood was added when anaerobes were tested), and each plate was inoculated with up to 21 strains using a multi-point inoculator (Denley) giving an inoculum size of $\sim 10^5$ bacteria. Incubation was at 37° for 24 h aerobically or 48 h anaerobically (using the GasPak system). The strains tested had all (except for the Oxford Staphylococcus) recently been isolated from clinical material. The "Gram-positive" group comprised 3 *Staphylococcus aureus,* 3 *Staphylococcus epidermidis* and 3 *Streptococcus faecalis*; the "Gram-negative" group consisted of 3 *Escherichia coli,* 3 *Klebsiella pneumoniae* and 3 *Pseudomonas aeruginosa*; "yeasts" were 3 *Candida albicans*; "anaerobes" were 5 *Clostridium difficile,* 2 *Bacteroides fragilis,* 1 *Veillonella sp.* and 1 *Clostridium perfringens.*

Results are shown in Table I in which the anti-neoplastic agents have been grouped according to the suggestions of Laurence (1973). It was not always possible to determine the precise minimum inhibitory concentration (MIC) for all compounds, as there was often too little material available; for this reason results have been expressed semi-quantitatively to enable an overall view to be obtained. Any compound showing ++ activity or greater was investigated further, and conventional MIC determined (Table II). It can be seen that mitomycin C was by far the most active compound.

The results show that the antimicrobial activity of anti-neoplastic agents is generally of a low order, but not confined to any one type of agent. The findings confirm and extend the fragmentary reports in the literature concerning mitomycin C, doxyrubicin, 5-fluorouracil, cytarabine and methotrexate (Grey & Hamilton-Miller 1977, Moody *et al.*, 1978, Michel *et al.*, 1979, Jacobs *et al.*, 1979, Wright & Matsen 1980) against a limited range of bacteria, and 5-fluorouracil, hydroxyurea, doxyrubicin, vinblastine, cyclophosphamide and cytarabine against *C. albicans* (Land *et al.*, 1980).

Following these results, studies on the antimicrobial action of anti-neoplastic agents might be more profitably confined to possible synergistic effects with antibiotics (Moody *et al.*, 1978) or to their possible mutagenic effects on bacteria.

I am most grateful to Mr A. Iliffe for assistance, and to The Royal Free Hospital Endowment Fund for support. I also acknowledge with gratitude numerous gifts of anti-neoplastic agents from many Drug Companies.

Received 11 October 1983; accepted 24 November 1983.

### References

GREY, D. & HAMILTON-MILLER, J.M.T. (1977). Trimethoprim-resistant bacteria: cross-resistance patterns. *Microbios,* **19,** 45.

JACOBS, J.Y., MICHEL, J. & SACKS, T. (1979). Bactericidal effect of combinations of antimicrobial drugs and antineoplastic antibiotics against *Staphylococcus aureus. Antimicr. Ag. Chemoth.,* **15,** 580.

LAND, G.A., HULME, K.L. & CHAFFIN, W.L. (1980). The effect of selected antineoplastic agents on the morphology of *Candida albicans* 5865. *Canad. J. Microbiol.,* **26,** 812.

LAURENCE, D.R. (1973). *Clinical Pharmacology* (4th edition). Edinburgh: Churchill Livingstone. p. 29.4.

© The Macmillan Press Ltd. 1984

**Table I** Activity of 21 anti-neoplastic agents against 4 groups of pathogenic micro-organisms, expressed in semi-quantitative terms

| Antineoplastic agent | Activity against indicated type of organism: Gram-positive bacteria | Gram-negative bacteria | Anaerobic bacteria | Yeasts |
|---|---|---|---|---|
| *Alkylating agents* | | | | |
| Carmustine | 0 | 0 | 0 | ± |
| Chlorambucil | 0 | 0 | ± | ± |
| Neoplatin | 0 | + | ND | 0 |
| No activity shown by busulphan, cyclophosphamide, dibromomannitol and melphalan. | | | | |
| *Anti-metabolites* | | | | |
| Aminopterin* | ++/± | ± | ++ | ± |
| Azathioprine | 0 | 0 | + | 0 |
| 5-fluorouracil* | +++/++ | ± | + | + |
| Methotrexate* | ++/± | 0 | 0 | ± |
| Thioguanine | ± | ± | ± | 0 |
| No activity shown by cytarabine, mercaptopurine. | | | | |
| *Inhibitors of cell division* | | | | |
| Etoposide | + | 0 | + | 0 |
| Vinblastine | ± | 0 | ND | ± |
| No activity shown by vincristine. | | | | |
| *Antibiotics* | | | | |
| Doxyrubicin | 0 | 0 | + | 0 |
| Mitomycin C | ++++ | ++ | ++++ | 0 |
| *Miscellaneous* | | | | |
| Dacarbazine | ± | ± | + | 0 |
| Hydroxyurea | 0 | ± | 0 | 0 |
| Procarbazine | ± | 0 | 0 | 0 |

Key: ++++ represents MIC in the range 0.01 – 0.1 $\mu g\,ml^{-1}$

+++ 0.1 – 1

++ 1 – 10

+ 10 – 100

± 100 – 1000

0 >1000

ND = not determined

*aminopterin, methotrexate and 5-fluorouracil showed significantly higher activity against *Strept faecalis* than against staphylococci, hence two scores in "Gram-positive" category.

**Table II** Minimum inhibitory concentrations (MIC) of four antineoplastic compounds against different groups of bacteria

| Compound | MIC against bacteria ($\mu g\,ml^{-1}$) Range of values observed: Gram-positive | | Gram-negative | Anaerobes |
|---|---|---|---|---|
| | *Staphylococci* | *Streptococcus faecalis* | | |
| Mitomycin C | 0.06 – 0.25 | 0.06 | 0.5 – 8 | 0.05 – 0.5 |
| 5-fluorouracil | 0.5 – 8 | 0.13 – 0.25 | ≥256 | 10 – 100 |
| Aminopterin | 256 – 1024 | 8 | 256 – 512 | 1 – 10 |
| Methotrexate | 64 – 1024 | 8 – 16 | >1024 | >100 |

MICHEL, J. JACOBS, J.Y. & SACKS, T. (1979). Bactericidal effect of combinations of antimicrobial drugs and antineoplastic antibiotics against Gram-negative bacilli. *Antimicr. Ag. Chemoth.*, **16,** 761.

MOODY, M.R. MORRIS, M.J., YOUNG, V.M., MOYE, L.A. SCHIMPFF, S.C., & WIERNIK, P.H. (1978). Effect of two cancer chemotherapeutic agents on the antibacterial activity of three antimicrobial agents. *Antimicr. Ag. Chemoth.*, **14,** 737.

WRIGHT, D.N. & MATSEN, J.M. (1980). Bioassay of antibiotics in body fluids from patients receiving cancer chemotherapeutic agents. *Antimicr. Ag. Chemoth.*, **17,** 417.